# Validation of the Korean version of the European Organization for Research and Treatment of Cancer brain cancer module (EORTC QLQ-BN20) in patients with brain tumors

**DOI:** 10.1186/1477-7525-11-145

**Published:** 2013-08-27

**Authors:** Yong Soon Shin, Jeong Hoon Kim

**Affiliations:** 1 Department of Nursing, Changwon National University, 92 Toechonro, Uichang-gu, Changwon, Gyeongnam 641-773, South Korea; 2 Department of Neurology Surgery, University of Ulsan College of Medicine, Asan Medical Center, Asanbyeongwon-gil 86, Songpa-gu, Seoul 138-736, South Korea

**Keywords:** Brain tumor, Health-related quality of life, QLQ-BN20, QLQ-C30, Validation, Korea

## Abstract

**Background:**

The European Organization for Research and Treatment of Cancer Quality of Life Brain Cancer Module has been translated into Korean, but to date, its reliability and validity have been evaluated in a pilot study alone. The European Organization for Research and Treatment of Cancer Quality of Life Core Questionnaire is, overall, a valid instrument to assess the health-related quality of life in Korean cancer patients, although its reliability and validity have not yet been evaluated in patients with brain tumors. This study aimed at evaluating the psychometric properties of these instruments in patients with brain tumors.

**Findings:**

The 2 instruments were used for 307 Korean patients with brain tumors. Multi-trait scaling confirmed the scale structure of the instruments with good item convergent and discriminant validity. The reliability was acceptable for all scales except for cognitive functioning and nausea and vomiting. The instruments could be used to distinguish between clinically distinct groups of patients.

**Conclusions:**

The study findings indicate that the instruments are valid and suitable for the assessment of the health-related quality of life in patients with brain tumors as well as in those with primary brain cancer.

## Background

Despite improvements, many treatments for brain tumors are not curative and these patients have a poor prognosis. In addition, such treatments may be neurotoxic and, thus, negatively affect patient health-related quality of life (HRQOL). Therefore, the use of well-validated instruments when assessing the HRQOL of patients with brain tumors is particularly important. Most HRQOL instruments were developed in English for a predominantly English-speaking population. The European Organization for Research and Treatment of Cancer brain cancer module (EORTC QLQ-BN20) has previously been tested in English-speaking patients and was shown to have adequate psychometric properties [1]. The QLQ-BN20 was translated into Korean by the EORTC group [2], and its reliability and validity have been evaluated, although only in a pilot study. The standard Korean version of EORTC Quality of Life Core Questionnaire (QLQ-C30) is, overall, a valid instrument for the assessment of HRQOL in Korean patients with breast, stomach, colon, and rectal cancers [3], although its reliability and validity have not yet been specifically evaluated in patients with brain tumors. Hence, the purpose of the current study was to examine the validity and reliability of the Korean version of the QLQ-BN20 and QLQ- C30 in patients with brain tumors.

## Methods

### Sample and setting

This study used a prospective, descriptive cross-sectional design in which the convenience sampling was performed at a tertiary-care university hospital in Seoul, Korea. To be included in the study, patients had to be aged >18 years, fluent in Korean, and diagnosed with a histologically verified brain tumor. Patients who had not undergone surgery or those with metastatic brain tumors were excluded. The study was approved by the institutional review board of Asan Medical Center. Written informed consent was obtained from all patients or their legally authorized representative.

### Instruments

The EORTC QLQ-BN20 is a 20-item questionnaire grouped into 4 domains and 7 single items [1], while the QLQ-C30 comprises a 30-item questionnaire. Raw scores were standardized by linear transformation so that the final scores were in the range of 0 –100. Higher scores on the global QOL and functional scales represent a better QOL, whereas high symptom scale scores indicate significant symptoms or greater difficulty.

### Statistical analysis

Multi-trait scaling was employed to examine the scale structure of the instruments. Item-scale correlations ≥ 0.40 were considered to validate item convergent. Item-scale discriminant validity was examined by comparing the correlation of each item with its own scale versus that with other scales. We expected a high correlation between items (2 standard errors) with their own scale rather than with other scales. The internal consistency reliability of the instruments was estimated using Cronbach’s alpha coefficient (α). Values of α ≥ 0.70 were considered acceptable for group comparisons.

The known group validities were tested by comparing the score of the Korean version of the QLQ-BN20 and QLQ-C30 between patient groups. First, we hypothesized that patients with a high performance status (Karnofsky performance scale [KPS] score > 70) would report lower levels of symptoms and a better QOL than patients with a low performance status (KPS score ≤ 70). Second, patients with glioma were expected to report a higher level of symptoms than patients with meningioma. Finally, patients who received adjuvant therapy (chemotherapy, radiation therapy, or both) were expected to report a higher level of future uncertainty than patients who underwent surgery alone.

Construct validity was then examined by calculating the correlations between the multi-item scales of the QLQ-BN20 and QLQ-C30. We expected moderate correlations between future uncertainty and emotional functioning, visual disorder and cognitive functioning, motor dysfunction and physical functioning, and communication deficit and social functioning (*r* > 0.40). We used SPSS 20.0 (SPSS Inc., Chicago, IL, USA) for data analysis. Values of *p* < 0.05 were considered statistically significant. Missing data were excluded from the analysis.

## Results

Of 350 invited patients, 317 (90.6%) agreed to participate, and a total of 307 (87.7%) useful questionnaires were analysed. The mean age was 47.95 ± 13.64 years (range, 18–81 years). Fifty-seven percent of the patients were women. Most patients were married (72%), and 37.5% had completed college or graduate school. Gliomas (39.7%) constituted the most frequent tumor type. More than half of the patients underwent surgery alone (Table 1).

**Table 1:** Socio-demographic and clinical characteristics (N=307)

**Variable**	**Mean** (**SD**, **range**) **or N** (%)
Age (years)	47.95 (13.64, 18–81)
Gender	
Female	175 (57.0)
Male	132 (43.0)
Education	
Middle school or less	77 (25.1)
High school	104 (33.9)
College	105 (34.2)
Graduate school	10 (3.3)
Nonresponse	11 (3.5)
Marital status	
Married	221 (72.0)
Single	53 (17.3)
Widowed/divorced	27 (8.8)
Nonresponse	6 (1.9)
KPS	
≤70	35 (11.4)
>70	272 (88.6)
Treatment modality	
Surgery only	177 (57.7)
Surgery plus CTx or RTx or both	130 (42.3)
Tumor type	
Glioma	122 (39.7)
Meningioma	107 (34.9)
Others	78 (25.4)

*KPS* Karnofsky performance scale, *CTx* chemotherapy, *RTx* radiation therapy.

### Scale reliability and scale structures

As shown Table 2, with the exception of the cognitive functioning (0.60) and nausea and vomiting scales (0.64), most of the multi-item scales of the QLQ-C30 and the all scales of the QLQ-BN20 met the minimal standard of reliability (α > 0.70).

**Table 2:** Scale reliability and structures

	**Mean** (**SD**)	**Cronbach**’**s alpha**	**Number of items**	**Item**-**own scale correlation**	**Item**-**other scale correlation**
**QLQ**-**C30**					
***Functional scales***					
Physical functioning	71.16 (24.94)	.87	5	0.55–0.90	0.16–0.55
Role functioning	69.74 (31.43)	.89	2	0.93–0.94	0.25–0.69
Emotional functioning	71.51 (23.81)	.87	4	0.79–0.87	0.27–0.56
Cognitive functioning	69.82 (25.80)	.60	2	0.77–0.87	0.23–0.55
Social functioning	71.73 (30.72)	.85	2	0.90–0.94	0.28–0.50
***Global quality of life***	54.19 (25.18)	.88	2	0.94–0.94	0.29–0.50
***Symptom****scales*/*items*					
Fatigue	37.42 (23.04)	.77	3	0.76–0.87	0.27–0.62
Nausea and vomiting	11.67 (17.53)	.64	2	0.66–0.94	0.19–0.38
Pain	25.11 (26.93)	.81	2	0.89–0.91	0.26–0.55
Dyspnea	20.37 (26.22)		1	1.00	0.30-0.51
Sleep disturbance	25.08 (31.61)		1	1.00	0.27–0.44
Appetite Loss	16.94 (24.90)		1	1.00	0.21–0.42
Constipation	17.10 (25.78)		1	1.00	0.14–0.25
Diarrhea	12.46 (22.57)		1	1.00	0.12–0.20
Financial difficulty	31.38 (35.19)		1	1.00	0.24–0.63
**QLQ**-**BN20** scales					
Future uncertainty	34.38 (23.67)	.80	4	0.72–0.84	0.21–0.47
Visual disorder	27.27 (28.22)	.83	3	0.73–0.90	0.19–0.36
Motor dysfunction	29.14 (28.92)	.85	3	0.81–0.85	0.25–0.49
Communication deficit	22.41 (27.82)	.90	3	0.84–0.92	0.18–0.50
**QLQ**-**BN20** single items					
Headache	33.44 (31.07)		1	1.00	0.30–0.41
Seizure	9.84 (23.06)		1	1.00	0.13–0.32
Drowsiness	43.32 (28.03)		1	1.00	0.26–0.42
Hair loss	21.57 (31.01)		1	1.00	0.00–0.26
Itchy skin	16.61 (25.05)		1	1.00	0.16–0.26
Weakness of legs	35.42 (33.27)		1	1.00	0.28–0.73
Bladder control	21.39 (30.88)		1	1.00	0.28–0.39

*QLQ*-*C30* Quality of Life Core Questionnaire, *QLQ*-*BN20* Quality of Life Questionnaire Brain Cancer Module.

The all-item scale of the QLQ-C30 correlated with its own scale with a value of *r* ≥ 0.50, corrected for overlap, and met the recommended psychometric standards. Item-scale correlations of the QLQ-BN20 indicated that each item had a stronger significant correlation with its own scale (range, 0.72–0.92) than with other scales (range, 0.18–0.50).

### Clinical validity

As hypothesized, patients with higher KPS scores had significantly better functioning and lower symptom scores on all of the QLQ-C30 and QLQ-BN20 multi-item scales than patients with lower KPS scores (*p* < 0.001–0.005, Table 3). Patients with glioma had significantly lower physical, cognitive, and social functioning scores as well as higher future uncertainty, motor dysfunction, and communication deficits than patients with meningioma (*p* < 0.001–0.02). Patients who underwent surgery plus adjuvant therapy reported lower functioning (*p* < 0.001–0.03) and poor QOL (*p* = 0.01), but higher future uncertainty (*p* = 0.02) and great communication deficits (*p* = 0.03) than those who underwent surgery alone. The 4 scales of the QLQ-BN20 were moderately correlated with some of the QLQ-C30 scales as hypothesized (*r* = −.410 – -.642, Table 4).

**Table 3:** **Mean scores** (**standard deviation**) **of clinically distinct groups**

	**Performance status**			**Tumor type**				**Treatment modality**		
	**KPS**≤**70**	**KPS**>**70**	p^*^	**Glioma**	**Meningioma**	**Others**	***p***^†^	**OP only**	**OP**+**adjuvant therapy**	p^*^
**QLQ**-**C30**										
PF	29.8 (27.4)	76.4(19.0)	<.001	66.2 (29.0)	75.1 (20.0)	73.7 (22.8)	.02^a^	73.8 (22.2)	67.6 (27.9)	.11
RF	27.3 (31.1)	74.9 (27.3)	<.001	64.5 (35.7)	74.5 (24.7)	71.5 (31.6)	.05	71.8 (27.8)	66.9 (35.7)	.84
EF	56.2 (26.6)	73.5 (22.7)	<.001	68.4 (24.8)	74.1 (20.6)	72.9 (26.0)	.18	71.2 (24.1)	71.9 (23.5)	.89
CF	42.9 (30.3)	73.3 (23.0)	<.001	64.1 (27.7)	73.2 (24.6)	74.2 (22.7)	.008^b^	72.3 (25.3)	66.4 (26.2)	.03
SF	36.3 (32.7)	76.2 (27.4)	<.001	63.6 (33.6)	81.0 (26.4)	71.9 (29.4)	<.001^c^	77.2 (28.2)	64.2 (32.5)	<.001
QOL	29.4 (22.3)	57.3 (23.8)	<.001	50.8 (26.3)	58.1 (23.9)	54.1 (24.6)	.09	57.3 (24.7)	50.0 (25.4)	.01
FA	56.5 (24.2)	35.0 (21.7)	<.001	40.4 (25.6)	34.2 (20.4)	37.3 (21.9)	.13	36.2 (21.6)	39.2 (24.8)	.24
NV	21.4 (25.1)	10.4 (15.9)	.005	13.9 (18.7)	9.0 (14.4)	11.8 (19.2)	.12	10.9 (17.9)	12.7 (17.0)	.05
PA	51.4 (29.8)	21.7 (24.6)	<.001	26.2 (29.6)	23.7 (24.0)	25.2 (26.6)	.79	25.8 (26.3)	24.2 (27.9)	.36
**QLQ**-**BN20**										
FU	47.1 (26.6)	32.7 (22.8)	.002	38.5 (24.2)	28.2 (21.1)	36.5 (24.7)	.003^d^	32.0 (23.8)	37.5 (23.3)	.02
VD	40.3 (31.4)	25.6 (27.4)	.002	22.3 (26.2)	30.6 (27.3)	30.5 (31.5)	.05	29.1 (29.4)	24.8 (26.4)	.23
MD	73.7 (29.4)	23.4 (23.3)	<.001	34.2 (32.6)	23.4 (23.0)	29.2 (28.9)	.02^e^	26.9 (26.3)	32.2 (32.0)	.33
CD	47.7 (34.5)	19.2 (25.2)	<.001	30.1 (31.4)	16.0 (23.4)	19.1 (24.7)	<.001^f^	19.3 (25.9)	26.6 (29.8)	.03

^*^p-value was calculated by Mann–Whitney *U* test.

^†^*p*-value was calculated by ANOVA.

^a, c^ Post-hoc test by Sheffe; glioma<meningioma.

^b^ Post-hoc test by Sheffe; glioma<meningioma, glioma<others.

^d, e, f^ Post-hoc test by Sheffe; glioma>meningioma.

*KPS* Karnofsky performance scale, *OP* operation, *QLQ*-*C30* Quality of Life Core Questionnaire, *PF* physical functioning, *RF* role functioning, *EF* emotional functioning, *CF* cognitive functioning, *SF* social functioning, *FA* fatigue, *NV* nausea and vomiting, *PA* pain, *QLQ*-*BN20* Quality of Life Questionnaire Brain Cancer Module, *FU* future uncertainty, *VD* visual disorder, *MD* motor dysfunction, *CD* communication deficit.

**Table 4:** **Correlations between the Korean versions of the QLQ**-**C30 and QLQ**-**BN20**

	**C30**-**PF**	**C30**-**RF**	**C30**-**EF**	**C30**-**CF**	**C30**-**SF**	**C30**-**QOL**
**BN20** - **FU**	-.475^*^	-.455^*^	-.634^*^	-.486^*^	-.534^*^	-.548^*^
**VD**	-.319^*^	-.340^*^	-.367^*^	-.447^*^	-.282^*^	-.348^*^
**MD**	-.642^*^	-.617^*^	-.499^*^	-.553^*^	-.532^*^	-.507^*^
**CD**	-.379^*^	-.396^*^	-.388^*^	-.511^*^	-.410^*^	-.378^*^

^*^ Correlation is significant at the 0.01 level (2-tailed).

*QLQ*-*C30* Quality of Life Core Questionnaire, *QLQ*-*BN20* Quality of Life Questionnaire Brain Cancer Module, *PF* physical functioning, *RF* role functioning, *EF* emotional functioning, *CF* cognitive functioning, *SF* social functioning, *QOL* quality of life; *FU* future uncertainty, *VD* visual disorder, *MD* motor dysfunction, *CD* communication deficit.

## Discussion

The validity and reliability of the EORTC QLQ-BN20 and QLQ-C30 have been shown in studies across various countries [4,5]. Here we present the results of a study using the Korean version of the QLQ-BN20 and QLQ-C30 in patients with brain tumors.

The internal consistency reliability coefficients of the QLQ-BN20 were high, although our subjects had various types of brain tumors. These reliability coefficients are higher than those reported in studies of the English [1] and other language versions [4,6]. Our results suggest that the QLQ-BN20 can be used to assess the HRQOL in patients with other brain tumors as well as in those with brain cancer.

Most of the item-subscale correlation coefficients in the QLQ-C30 met the required convergent and discriminant validity standards. This has not always been the case; for example, the structures of the Mexican-Spanish and Greek versions of the QLQ-C30 had weaknesses with respect to both their assessment of the cognitive functioning scale and the nausea and vomiting item [7-10]. In this study, we confirmed that the cognitive functioning scale and the nausea and vomiting item were improved as shown in an earlier study of Korean patients with cancer [3].

Most scores of the 2 instruments could clearly distinguish between patient groups according to performance status as shown in earlier studies [4,6,10,11]. The second hypothesis, that the 2 instruments could distinguish patients with glioma from those with meningioma, was partially supported. A Chinese group [12] reported that most scores of the EORTC-C30 were able to distinguish among patients with different brain tumor types. However, they did not compare differences of functioning, QOL and symptoms among specific tumor types (e.g. glioma vs meningioma) using a post-hoc test.

The third hypothesis, that the adjuvant treatment group had greater future uncertainty than the group who underwent surgery alone, was supported. This may have been because the patients who received adjuvant therapy after surgery perceived themselves to have more severe disease than those who underwent surgery alone.

Finally, we confirmed that the construction validities of the QLQ-BN20 and QLQ-C30 were acceptable, with correlation coefficients values of *r* > 0.40. These results are similar to those of the previous validation studies of the QLQ-BN20 and QLQ-C30 conducted for patients speaking English [1], Persian [6], and 15 other languages [4].

The present study had some limitations. First, there might have been sampling bias since all participants were recruited from a single hospital and accurate sample size for this study was not calculated. Second, we did not include responsiveness analysis because of the lack of follow-up data. Therefore, these results should be carefully interpreted and implemented.

In conclusion, our findings suggest that the Korean versions of the EORTC QLQ-BN20 and QLQ-C30 are clinically valid and reliable for measuring HRQOL and are suitable for use in both clinical practice and clinical studies, involving Korean patients with brain tumors.

## Abbreviations

EORTC QLQ-BN20: European organization for research and treatment of cancer quality of life questionnaire brain cancer module; HRQOL: Health-related quality of life; EORTC QLQ-C30: Quality of life core questionnaire; QOL: Quality of life; KPS: Karnofsky performance scale.

## Competing interests

The authors confirm that no research funding or any support was received for this study and declare that they have no financial or other conflicts of interest related to this research and its publication.

## Authors’ contributions

SYS and KJH designed the study, KJH collected the data, and both SYS and KJH interpreted the data and revised the manuscript. SYS managed the statistical analyse. Both authors read and approved the final manuscript.
